# Placental extract suppresses differentiation of 3T3-L1 preadipocytes to mature adipocytes via accelerated activation of p38 MAPK during the early phase of adipogenesis

**DOI:** 10.1186/s12986-019-0361-8

**Published:** 2019-05-20

**Authors:** Yusuke Ando, Fumiaki Sato, Hazuki Fukunaga, Yusuke Iwasaki, Yoshihiko Chiba, Masahiko Tebakari, Yuki Daigo, Junichi Kawashima, Junzo Kamei

**Affiliations:** 1 Global Research Center for Innovative Life Science, Hoshi University School of Pharmacy and Pharmaceutical Sciences, 2-4-41 Ebara, Shinagawa-ku, Tokyo, 142-8501 Japan; 2 Laboratory of Analytical Pathophysiology, Division of Pharmacy Professional Development and Research, Hoshi University School of Pharmacy and Pharmaceutical Sciences, 2-4-41 Ebara, Shinagawa-ku, Tokyo, 142-8501 Japan; 3 Laboratory of Biopharmaceutics Analytical Science, Hoshi University School of Pharmacy and Pharmaceutical Sciences, 2-4-41 Ebara, Shinagawa-ku, Tokyo, 142-8501 Japan; 4 Department of Physiology and Molecular Sciences, Hoshi University School of Pharmacy and Pharmaceutical Sciences, 2-4-41 Ebara, Shinagawa-ku, Tokyo, 142-8501 Japan; 5 Pharmaceutical Research Laboratory, Snowden Co., Ltd., 793 Futatsumiya, Nishi-ku, Saitama, 331-0065 Japan; 6 R&D Merchandising Division, Snowden Co., Ltd., 3-7-16 Iwamoto-cho, Chiyoda-ku, Tokyo, 101-0032 Japan

**Keywords:** Placental extract, 3T3-L1, Adipogenesis, Obesity, p38 MAPK, PPARγ

## Abstract

**Background:**

Adipogenesis, the process of preadipocyte differentiation to mature adipocytes accompanied by accumulation of intracytoplasmic lipid droplets, is regulated by various genetic and environmental factors, and closely associated with the development of obesity. Numerous recent studies suggest that some bioactive peptides and proteins derived from animals, and chemical compounds isolated from plants may be useful for prevention and treatment of obesity and obesity-related chronic diseases. In the present study, we examined the broad spectrum of effects of placental extract, with a focus on the influence of placental extract on adipogenesis.

**Method:**

We cultured 3T3-L1 cells, which are widely used as a model of white preadipocytes, under differentiation conditions in the presence of porcine placental extract (PPE) for 8 days, and then stained the lipid droplets accumulated in the cytoplasm with Oil Red O. We also analyzed the effects of PPE on the mitogen-activated protein kinases (MAPKs) signaling, mitotic clonal expansion (MCE) and gene expressions associated with 3T3-L1 differentiation.

**Results:**

When we cultured 3T3-L1 cells with PPE under differentiation conditions, the accumulation of lipid droplets and expression of adipocyte differentiation marker genes (*Cebpa*, *Pparg*, *Slc2a4*, *Fasn* and *Adipoq*) were dramatically attenuated. The suppressive activity of PPE against adipogenesis was heat-stable and recovered in a low-molecular-weight fraction after ultrafiltration (< 3 kDa) and gel-filtration chromatography (fraction No. 9). We also found that the suppressive activity of PPE affected the early phase of adipocyte differentiation (Days 0–2) without influencing the expression levels of C/EBPβ and C/EBPδ. The PPE and fraction No. 9 obtained from gel-filtration chromatography both promoted mitotic clonal expansion of 3T3-L1 while accelerating p38 MAPK phosphorylation. In addition, SB203580, a p38 MAPK inhibitor, partially restored the accumulation of lipid droplets and the expression of adipocyte differentiation marker genes that were suppressed by fraction No. 9.

**Conclusion:**

These results indicate that PPE suppresses the differentiation of preadipocytes via accelerated activation of p38 MAPK during the early phase of adipogenesis, suggesting PPE or its functional component could be a potential therapy for treating obesity.

**Electronic supplementary material:**

The online version of this article (10.1186/s12986-019-0361-8) contains supplementary material, which is available to authorized users.

## Introduction

Obesity is one of the biggest health problems in the world today, not least because it leads to diseases such as diabetes, hypertension, fatty liver, cardiovascular disorders and cancers [1–4]. Various factors are known to contribute to the development of obesity, and a number of therapeutic and preventive approaches have been suggested [5, 6]. Since adipogenesis, the process of preadipocyte differentiation to mature adipocytes accompanied by lipid storage, is a particularly key process in obesity, controlling adipocytes—such as by suppressing adipocyte hypertrophy and hyperplasia—has been a major focus in the drug discovery process [7–9].

The effect of polyphenols, including resveratrol (3, 4′, 5-trihydroxystilbene), green tea catechins (GTC), epigallocatechin gallate (EGCG) and curcumin, on the prevention of obesity and obesity-related chronic diseases has recently been discussed [10–16]. In addition to the polyphenols, some sesquiterpenes and steroid metabolites have also been reported to exhibit anti-obesity effects [17, 18]. Moreover, globin peptide (Val-Val-Tyr-Pro), isolated from a globin digested with acidic protease, also inhibits adipogenesis [19], indicating that some bioactive peptides and proteins, and not only chemical compounds isolated from plants, may be useful for obesity prevention and treatment.

The placenta, an organ that connects the developing fetus to the uterine wall, plays crucial roles for the transfer of oxygen and nutrients to the fetus from the mother’s blood, and waste products to the mother’s blood from the fetus [20]. Since the placenta contains various nutrients including amino acids, nucleic acids, minerals, vitamins, hormones and some growth factors, it has been used in traditional medicines for fatigue, menopausal symptoms, skin whitening and antiaging from ancient times to the present [21–26]. Recent studies, in fact, have demonstrated that a hydrolyzed extract of the placenta, known simply as “placental extract”, has beneficial effects such as amelioration of non-alcoholic steatohepatitis (NASH) and other forms of liver regeneration, increased proliferation of skin fibroblasts and marrow cells, and anti-inflammatory and anti-oxidant activities [27–30]. In the present study, we examined the broad spectrum of effects of placental extract, with a focus on the influence of placental extract on adipogenesis. Our results strongly suggested that placental extract suppresses preadipocyte differentiation to mature adipocytes via accelerated activation of p38 MAPK during the early phase of adipogenesis, and could contribute to a solution to the global epidemic of obesity.

## Materials and methods

### Reagents, antibodies and cell culture

D-MEM/Ham’s F-12, 3-isobutyl-1-methylxanthine (IBMX), dexamethasone (DEX) and insulin were purchased from FUJIFILM Wako Pure Chemical Corporation (Osaka, Japan). Resveratrol was a product of Tokyo Chemical Industry (Tokyo). Newborn calf serum (NBCS) and fetal bovine serum (FBS) were purchased from Thermo Fisher (Waltham, MA) and Biosera (Boussens, France), respectively. SB203580 was purchased from Merck Millipore (Darmstadt, Germany). Oil Red O and anti-β-Actin antibody were purchased from Sigma-Aldrich (St. Louis, MO). Antibodies against C/EBPβ (H-7) and C/EBPδ (C-6) were products of Santa Cruz Biotechnology (Dallas, TX). Antibodies against p38 MAPK (D13E1), phospho-p38 MAPK (Thr180/Tyr182) (D3F9), ERK1/2 (137F5) and phospho-ERK1/2 (Thr202/Tyr204) (D13.14.4E) were purchased from Cell Signaling Technology (Danvers, MA). Oligonucleotides were supplied by FASMAC (Kanagawa, Japan).

3T3-L1 cells were supplied by the Riken Cell Bank (Tsukuba, Japan) and cultured with D-MEM/Ham’s F-12 medium supplemented with 10% heat-inactivated NBCS at 37 °C under humidified 5% CO_2_.

### Preparation of the porcine placental extract (PPE)

The porcine placental extract (PPE) used in this study was produced by Snowden (Tokyo) using a combination of fermentation and proteolysis as previously described [27]. Briefly, the porcine placenta was obtained as afterbirth and was fermented with yeast (*Zygosaccharomyces* sp.) and lactic acid bacteria (*Pediococcus* Sp.) for 1 day. After the supernatant was collected, the residual material was subjected to proteolysis to obtain the hydrolyzed supernatant. The supernatants were combined and lyophilized to afford the PPE powder. We used mainly this fermented PPE in the present study and also used another type of PPE that was produced by combining proteolysis and heat treatment, which has not been passed the fermented process, as shown in Additional file 2: Figure S2 [30]. The PPE powder was dissolved with ultrapure water at the concentration of 10 mg/mL with sonication for 15 min, followed by mixing by inverting and additional sonication for 15 min. After centrifugation at 1500 x g for 3 min, the supernatant was lyophilized, and the resultant pellet was dissolved with ultrapure water at a concentration of 100 mg/mL at 4 °C for 16 h on a rotator. After centrifugation at 1500 x g for 10 min, the supernatant was filtered with a membrane (0.22 μm pore) and stored at -20 °C until use. The ultrafiltration of PPE was performed using an Amicon Ultra-2 mL 3 K (Merck Millipore).

### Differentiation of 3T3-L1 preadipocytes to mature adipocytes

3T3-L1 cells were seeded on a 0.1% gelatin-coated 12-well plate and cultured with D-MEM/Ham’s F-12 supplemented with 10% NBCS until reaching confluence, followed by culturing with D-MEM/Ham’s F-12 supplemented with 10% FBS for 48 h (day 0). After incubation for 48 h with D-MEM/Ham’s F-12 supplemented with 10% FBS and IDM (0.5 mM IBMX, 0.25 μM DEX and 5 μg/mL insulin) (day 2), the medium was changed every 48 h with D-MEM/Ham’s F-12 supplemented with 10% FBS and 5 μg/mL insulin until day 8. Since resveratrol has been shown to exhibit anti-obesity effects such as inhibition of preadipocyte differentiation to mature adipocytes, decrease of adipocyte proliferation, induction of adipocyte apoptosis, inhibition of lipogenesis and promotion of lipolysis, we used resveratrol as a positive control in the present study [10, 14–16].

### WST assay

Cytotoxicity against 3T3-L1 cells and IDM-induced proliferation of 3T3-L1 cells were evaluated by WST assay using a Cell Counting Kit-8 (Dojindo Laboratories, Kumamoto, Japan). The procedures were performed according to the manufacturer’s protocols.

### Oil red O staining

Cells in a 12-well plate were washed twice with PBS and fixed with 10% formaldehyde in PBS for 10 min at room temperature. After washing with PBS, the cells were washed with 60% isopropanol and stained with 0.18% Oil Red O in 60% isopropanol for 20 min at room temperature, followed by a final washing with 60% isopropanol and then a final washing with PBS. The resultant stained lipid droplets were imaged by bright field microscopy, and then the dye in the cells was extracted with isopropanol and quantified by measuring its absorbance at 492 nm with a microplate reader (MTP-450; Corona Electric, Ibaraki, Japan).

### RT-qPCR

Total RNA was extracted from cells using RNAzol RT Reagent (Molecular Research Center, Cincinnati, OH), and cDNA was synthesized from the total RNA using a ReverTra Ace qPCR RT Master Mix with gDNA Remover (TOYOBO, Osaka, Japan). The procedures were performed according to the respective manufacturer’s protocols. The qPCR reactions were conducted in an Applied Biosystems StepOne system (Life Technologies) with KAPA SYBR FAST qPCR Kit Master Mix (2X) ABI Prism (KAPA Biosystems, Boston, MA). All samples were analyzed in triplicate and quantified by the relative standard curve method using the gene expressions of *Rplp0* as an internal control. The sequences of the primer pairs used in this study are listed in Table 1.

**Table 1:** Oligodeoxynucleotide primers used in the RT-qPCR experiments

Gene	Forward (5′-3′)	Reverse (5′-3′)
*Rplp0*	AGATTCGGGATATGCTGTTGGC	TCGGGTCCTAGACCAGTGTTC
*Cebpa*	CAAGAACAGCAACGAGTACCG	GTCACTGGTCAACTCCAGCAC
*Pparg*	TCGCTGATGCACTGCCTATG	GAGAGGTCCACAGAGCTGATT
*Slc2a4*	GTGACTGGAACACTGGTCCTA	CCAGCCACGTTGCATTGTAG
*Fasn*	GGAGGTGGTGATAGCCGGTAT	TGGGTAATCCATAGAGCCCAG
*Adipoq*	TGTTCCTCTTAATCCTGCCCA	CCAACCTGCACAAGTTCCCTT
*Cebpb*	AAGCTGAGCGACGAGTACAAGA	GTCAGCTCCAGCACCTTGTG
*Cebpd*	TTCAGCGCCTACATTGACTC	GCTTTGTGGTTGCTGTTGAAG
*Cidea*	ATCACAACTGGCCTGGTTACG	TACTACCCGGTGTCCATTTCT
*Pgc1a*	CATTTGATGCACTGACAGATGGA	GTCAGGCATGGAGGAAGGAC
*Prdm16*	GACATTCCAATCCCACCAGA	CACCTCTGTATCCGTCAGCA

### Gel-filtration chromatography

A 100 mg/mL aliquot of PPE was separated by gel-filtration chromatography on a Superdex peptide 10/300 GL column (GE Healthcare, Marlborough, MA) equilibrated with 5-fold diluted phosphate-buffered saline (PBS). The elution was performed with 5-fold diluted PBS at a flow rate of 0.5 mL/min at room temperature, and fractions of 2 mL were collected using an AKTA pure 25 system (GE Healthcare). The pooled fractions were lyophilized, and the pellets were dissolved in one-fifth of the original volume of ultrapure water. The resultant fractions were diluted 1:100 with the culture medium and used for the experiments in the present study.

### Western blotting

Cells were lysed in 1 × SDS sample buffer (50 mM Tris-HCl, 1% SDS, 5% glycerol, 0.01% bromophenol blue, pH 6.8). The resultant cell lysates were sonicated and subjected to SDS-polyacrylamide gel electrophoresis (PAGE). SDS-PAGE and western blotting were conducted as described previously [31].

### Cell cycle analysis

Cell cycle analysis was performed as described previously [32]. In brief, cells were detached from the plate using Accutase (Innovative Cell Technologies, San Diego, CA), washed with PBS and fixed with 70% ethanol at -20 °C for 16 h. After washing twice with PBS, the cells were stained with 25 μg/mL propidium iodide (BioLegend, San Diego, CA) and analyzed using FACSVerse (BD Biosciences, Franklin Lakes, NJ). Data analysis was performed with FCS Express 4 (De Novo Software, Glendale, CA).

### Data analysis

Data analysis was performed with GraphPad Prism 5.0 (GraphPad Software, San Diego, CA). All data analyses were conducted using one-way analysis of variance (ANOVA) with Tukey-Kramer post hoc test, and values of *p* < 0.05 were considered statistically significant.

## Results

### PPE suppressed the accumulation of lipid droplets in 3T3-L1 cells

**Fig. 1:**
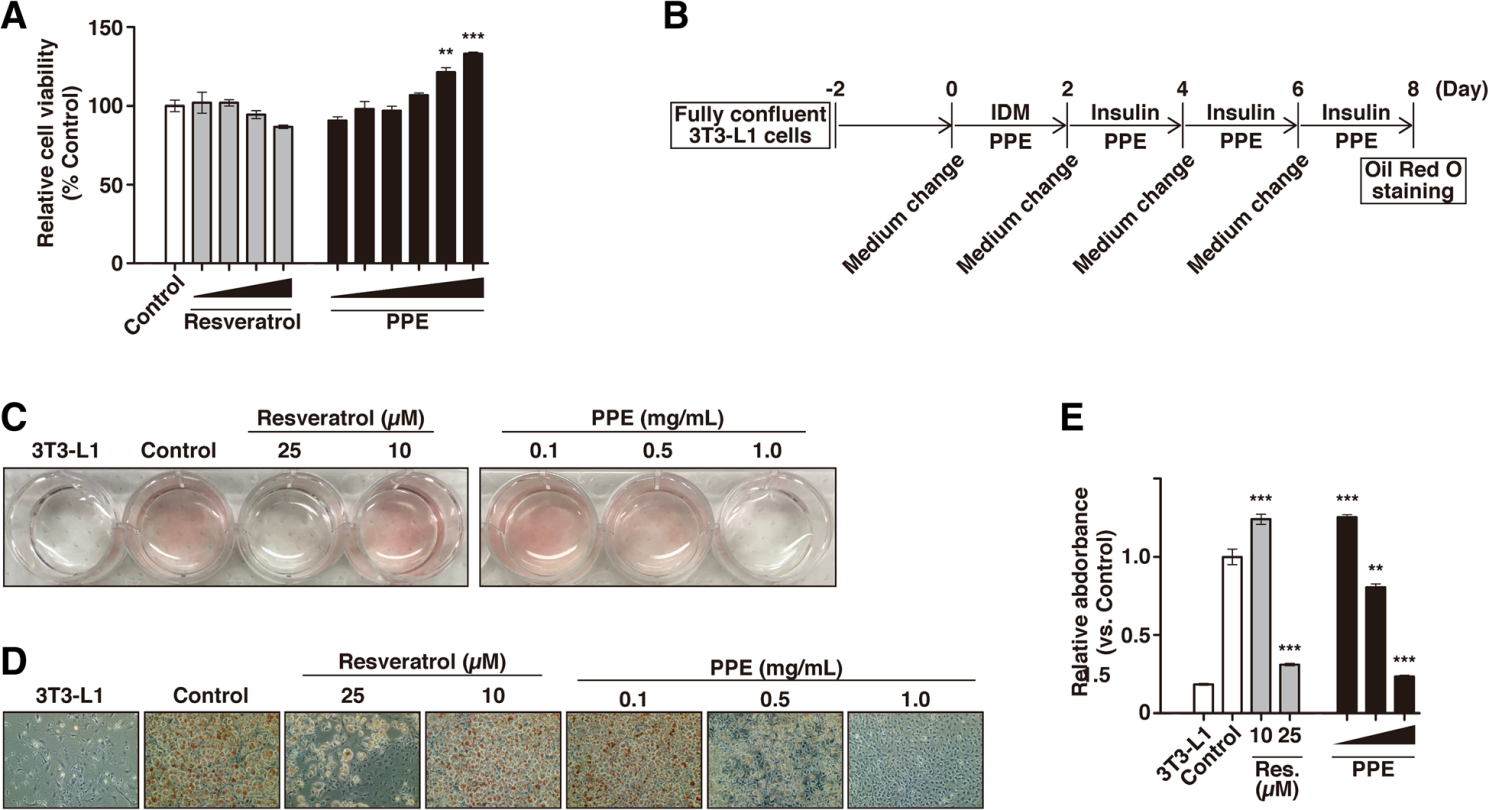
Decreased accumulation of lipid droplets in response to PPE in differentiated 3T3-L1 cells. **a** The cytotoxicities of PPE (1.0, 0.5, 0.25, 0.125, 0.0625 and 0.03125 mg/mL) and resveratrol (100, 50, 25 and 10 μM) against 3T3-L1 cells were analyzed by WST assay. The data are represented as relative cell viabilities compared to 3T3-L1 cells cultured without PPE (*Control*). **b** Time schedule of the culture with PPE during the cellular differentiation of 3T3-L1 cells. 3T3-L1 cells that reached confluence were cultured with or without PPE (1.0, 0.5 and 0.1 mg/mL) and IDM for 2 days. The medium was changed every 48 h to fresh medium containing 5 μg/mL insulin with or without PPE until day 8, followed by staining with Oil Red O. **c** The culture plate was photographed after staining. **d** Lipid droplets in differentiated 3T3-L1 cells cultured with PPE (1.0, 0.5 and 0.1 mg/mL) were stained with Oil Red O and imaged by bright field microscopy. **e** Stained lipid droplets were extracted with isopropanol and quantified by measuring the absorbance at 492 nm. Experiments were performed in triplicate, and the data are presented as the mean ± SEM (*n* = 3). **p < 0.01, ***p < 0.005 vs. *Control*. Experiments were repeated at least seven times, and representative results are shown

We investigated the effects of PPE on adipogenesis, including by comparing the effects of PPE and resveratrol, a well-known anti-obesity polyphenol. We first confirmed the cytotoxicity of PPE against 3T3-L1 cells, which are widely used as a model of white preadipocytes. We cultured 3T3-L1 cells with various concentrations of PPE (0.03125–1.0 mg/mL) for 48 h and then measured the cell viabilities by WST assay. The viabilities of the cells cultured with PPE were increased in a concentration-dependent manner compared to those of the control 3T3-L1 cells (Fig. 1a), suggesting that PPE had no cytotoxic effects on the 3T3-L1 cells after 48 h of culture. Resveratrol also had no cytotoxic effects at the concentrations used in this study (10–100 μM) (Fig. 1a).

We next analyzed the effects of PPE on the accumulation of lipid droplets of 3T3-L1 cells. Since it has been reported that resveratrol at concentrations of 10 μM or less has no anti-obesity effects on 3T3-L1 cells [10], we used 10 μM and 25 μM resveratrol as a negative and positive control, respectively. In agreement with the previous report, we found that 25 μM resveratrol, but not 10 μM resveratrol, suppressed the accumulation of lipid droplets in this study (Fig. 1c, d, and e). We cultured 3T3-L1 cells under differentiation conditions in the presence of PPE for 8 days, and then stained the lipid droplets accumulated in the cytoplasm with Oil Red O (Fig. 1b). 3T3-L1 cells stored the lipid droplets, which were associated with the differentiation to mature adipocytes, whereas the accumulation on cells cultured with PPE was decreased in a concentration-dependent manner (Fig. 1c and d). PPE at 1 mg/mL suppressed the accumulation of lipid droplets completely, and appeared to have no influence on cell morphology or viability (Fig. 1d). The decreased accumulation of lipid droplets on cells treated with PPE was also confirmed by measuring the absorbance of extracted Oil Red O (Fig. 1e).

### PPE suppressed the increased expression of adipocyte differentiation markers

**Fig. 2:**
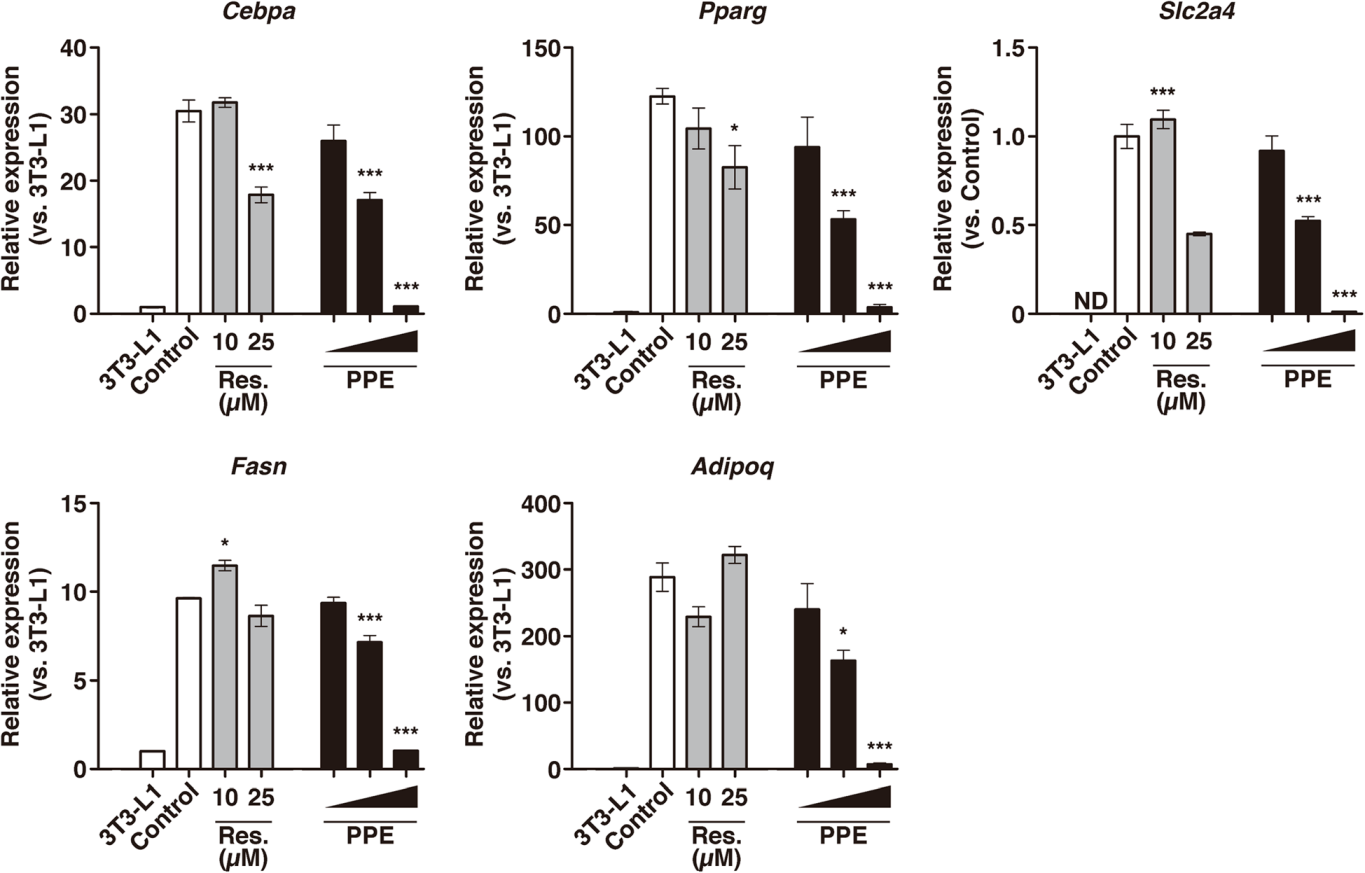
Suppression of the differentiation of preadipocytes to mature adipocytes by PPE. The gene expression of adipocyte differentiation markers, *Cebpa*, *Pparg*, *Slc2a4*, *Fasn* and *Adipoq*, in 3T3-L1 cells cultured with PPE (1.0, 0.5 and 0.1 mg/mL) under differentiation conditions were analyzed on day 8 by RT-qPCR. *Control* represents cells cultured without PPE, and *3T3-L1* represents cells cultured without either PPE or differentiation-inducing agents. The gene expressions of *Cebpa*, *Pparg*, *Fasn* and *Adipoq* in 3T3-L1 cells cultured with PPE are presented relative to the value in *3T3-L1* cells, and that of *Slc2a4* is presented relative to the value in *Control* cells. Experiments were performed in triplicate, and the data are presented as the mean ± SEM (n = 3). ND, not detected; *p < 0.05, ***p < 0.005 vs. *Control*. Experiments were repeated three times, and representative results are shown

We next examined the effects of PPE on the gene expressions of adipocyte differentiation markers (*Cebpa*, *Pparg*, *Slc2a4*, *Fasn* and *Adipoq*) by RT-qPCR. Differentiated 3T3-L1 cells have been shown to exhibit prominent increases in the expression of these genes relative to undifferentiated 3T3-L1 cells [33, 34]. In our experiments, PPE suppressed these gene expressions associated with 3T3-L1 differentiation in a concentration-dependent manner. At a PPE concentration of 1 mg/mL, these gene expressions were found to be suppressed at the same level as in undifferentiated 3T3-L1 cells (Fig. 2). We also analyzed the effects of PPE on the gene expressions of brown/beige adipocyte differentiation markers (*Cidea*, *Pgc1a* and *Prdm16*) [35]. We found that PPE suppressed these gene expressions in a concentration-dependent manner, suggesting that the suppression of the accumulation of lipid droplets in 3T3-L1 cells by PPE was unlikely to be due to the induction of thermogenic program of adipocytes (Additional file 1: Figure S1).

### The suppressive activity of PPE against adipocyte maturation was fractionated by gel-filtration chromatography

**Fig. 3:**
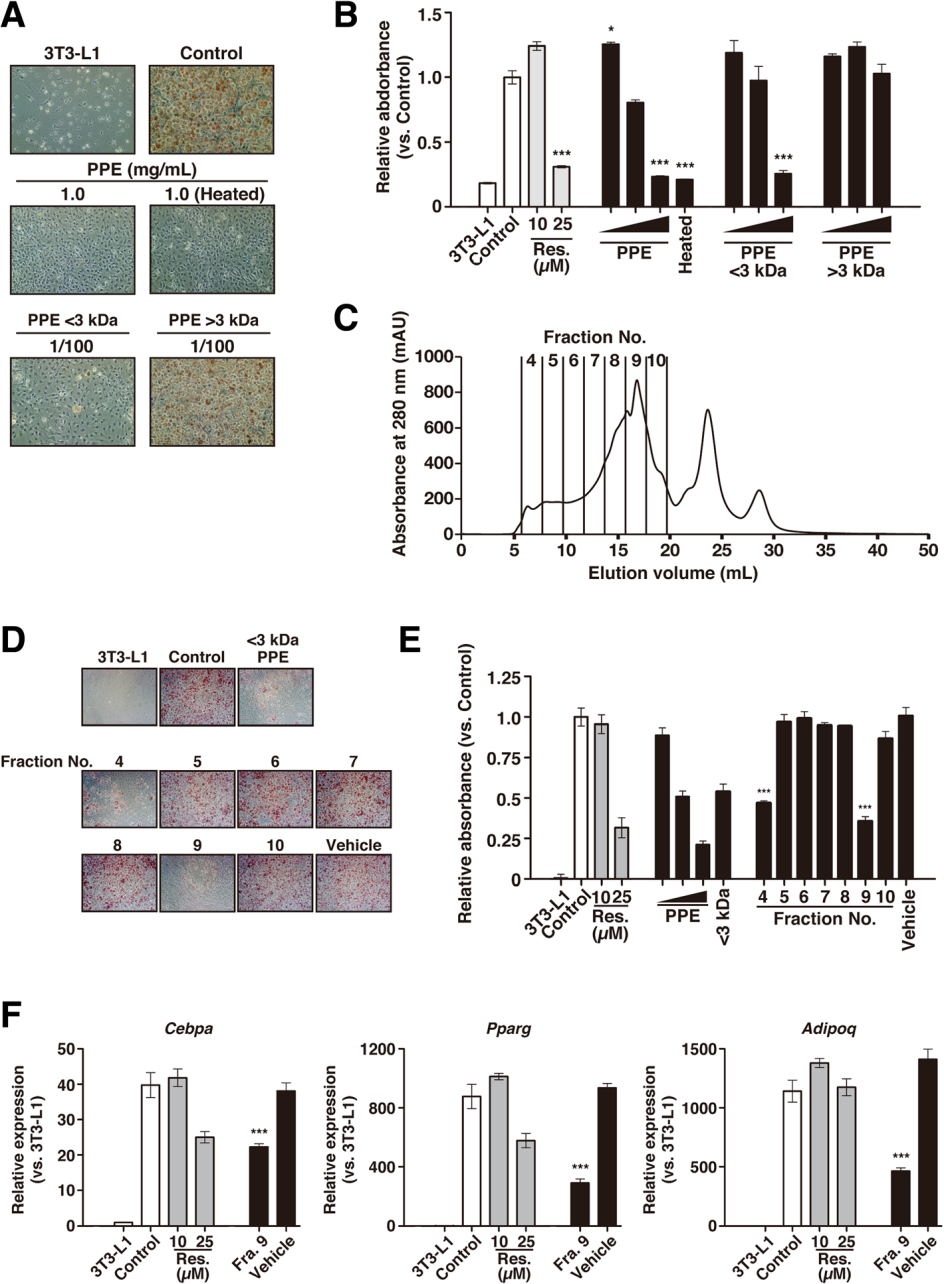
Suppressive activity of PPE separated by ultrafiltration and gel-filtration chromatography for adipogenesis. **a** 3T3-L1 cells were cultured under differentiation conditions with or without each ultrafiltrated fraction, < 3 kDa or > 3 kDa separated from PPE using an Amicon. After 8 days of differentiation culture, the cells were stained with Oil Red O, and lipid droplets in the cells were imaged by bright field microscopy. Each fraction was diluted with the culture medium at 1:100. **b** Stained lipid droplets were extracted with isopropanol and quantified by measuring the absorbance at 492 nm. **c** PPE was fractionated by gel-filtration chromatography on a Superdex peptide 10/300 GL column. The fraction numbers are shown on the chromatogram (fractions No. 4–10). **d** 3T3-L1 cells were cultured under a differentiation condition with or without each fraction (fractions No. 4–10) until day 8. After the cells were stained with Oil Red O, lipid droplets in the cells were imaged by bright field microscopy. **e** Stained lipid droplets were extracted with isopropanol and quantified by measuring the absorbance at 492 nm. **f** The gene expressions of adipocyte differentiation markers (*Cebpa*, *Pparg* and *Adipoq*) in 3T3-L1 cells cultured with fraction No. 9 under a differentiation condition were analyzed on day 8 by RT-qPCR. *Control* represents cells cultured without fraction No. 9, and the *3T3-L1* represents cells cultured without either fraction No. 9 or differentiation-inducing agents. *Vehicle* represents the cells cultured with PBS instead of each fraction. The gene expressions in 3T3-L1 cells cultured with fraction No. 9 are presented relative to the value in *3T3-L1*. Experiments were performed in triplicate, and the data are presented as the mean ± SEM (n = 3). *p < 0.05, ***p < 0.005 vs. *Control*. Experiments were repeated at least three times, and representative results are shown

To elucidate which components of PPE possessed the suppressive activity, we first separated PPE by ultrafiltration using an Amicon membrane into two fractions, the < 3 kDa and > 3 kDa fractions. Each fraction was then assayed to determine its level of suppressive activity against the accumulation of lipid droplets of 3T3-L1 cells. The < 3 kDa fraction showed significant suppressive activity against adipogenesis, whereas the > 3 kDa fraction did not (Fig. 3a and b). On the other hand, when we prepared a heated sample of PPE (heating at 100 °C for 30 min), we found that the suppressive activity was still preserved (Fig. 3a). We also obtained the same results using PPE solutions prepared by combining proteolysis and heat treatment (Additional file 2: Figure S2) [30], suggesting that PPE exhibited marked suppressive activity against adipogenesis regardless of the method of preparation. We next fractionated the PPE by gel-filtration chromatography on a Superdex peptide 10/300 GL column (Fig. 3c). When we used seven fractions (fractions No. 4–10) and assayed for the suppressive activity against adipogenesis, we found that fractions No. 4 and 9 showed significant suppressive activity against the accumulation of lipid droplets of 3T3-L1 cells (Fig. 3d and e). Since fraction No. 4 was equivalent to void volume, the substance that exhibited the suppressive activity against adipogenesis in fraction No. 4 was likely to be aggregations of the component at the fraction No. 9. Moreover, fraction No. 9 suppressed the gene expressions of adipocyte differentiation markers (*Cebpa*, *Pparg* and *Adipoq*) (Fig. 3f).

### PPE suppressed the differentiation of 3T3-L1 cells in the early phase of adipocyte maturation

**Fig. 4:**
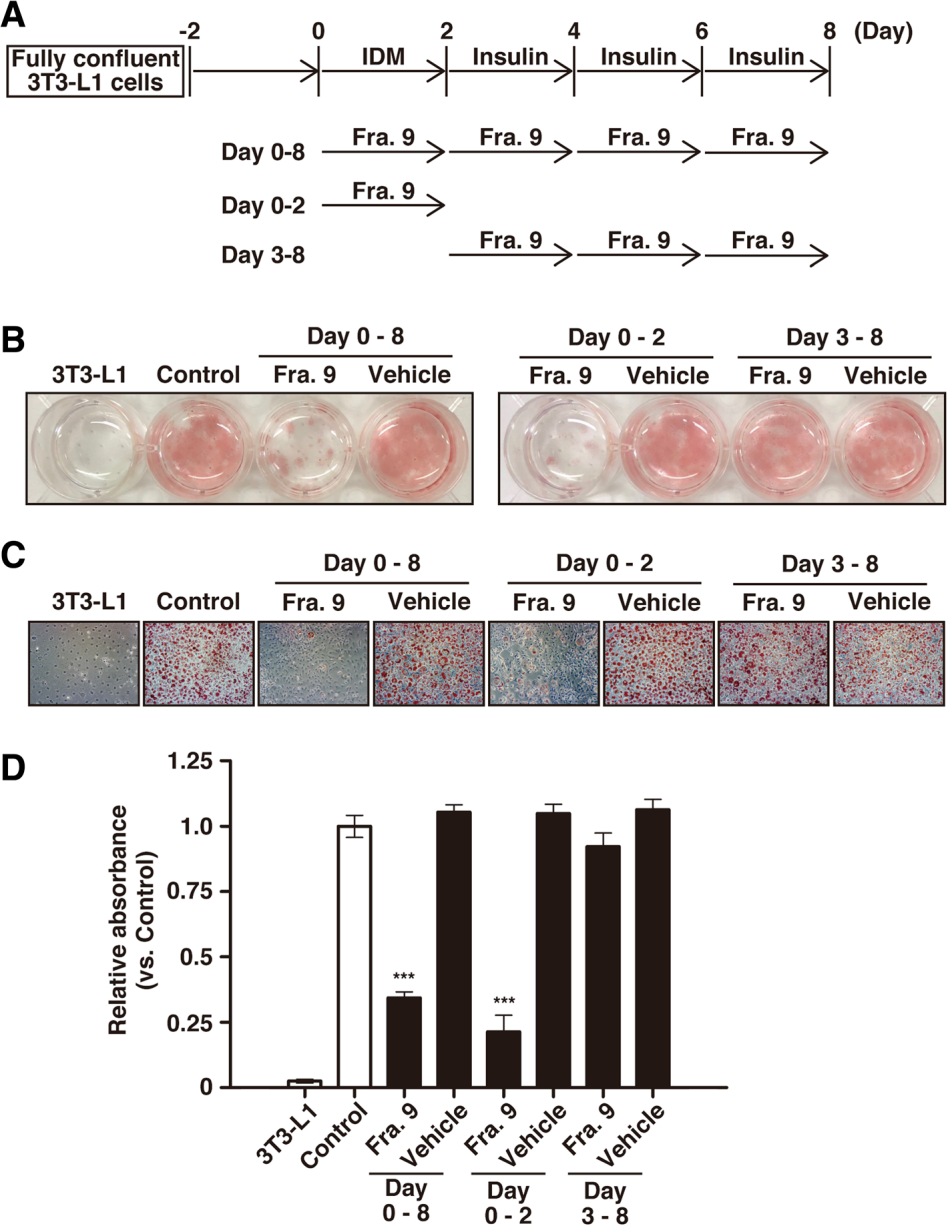
The suppressive activity of PPE against adipogenesis in the early phase of adipocyte maturation. **a** Time schedule for the culture with fraction No. 9 during the cellular differentiation of 3T3-L1 cells. 3T3-L1 cells which reached confluence were cultured with IDM for 2 days. The medium was changed every 48 h to fresh medium containing insulin until day 8. Fraction No. 9 was added during days 0–8, days 0–2 or days 3–8. After the cells were stained with Oil Red O, the culture plate was photographed (**b**) and lipid droplets in the cells were imaged with a bright field microscope (**c**), and then the extracted Oil Red O was quantified by measuring its absorbance at 492 nm (**d**). *Vehicle* represents the cells cultured with PBS instead of fraction No. 9. Experiments were performed in triplicate, and the data are presented as the mean ± SEM (n = 3). ***p < 0.005 vs. *Control*. Experiments were repeated at least three times, and representative results are shown

It is well known that various morphologic and genetic changes occur during adipocyte maturation, and the adipogenesis of the cells is divided into two phases, an early phase from day 0 to day 2 and a late phase from day 3 to day 8 [36, 37]. In order to examine the effects of PPE on the two phases, we added fraction No. 9 to 3T3-L1 cultures under differentiation conditions during day 0 to day 2 (early phase), day 3 to day 8 (late phase) or day 0 to day 8 (early and late phase) (Fig. 4a). The addition of fraction No. 9 to 3T3-L1 cultures under differentiation conditions during the early phase resulted in marked suppressive activity against adipogenesis, similar to the level of anti-adipogenesis activity induced by addition of fraction No. 9 during the early and late phase (Fig. 4b, c and d). On the other hand, when the fraction was added only during the late phase there was no suppressive activity at all. We also observed quite similar results when using PPE (data not shown). These findings suggest that the suppression of adipocyte maturation by PPE was due to the inhibition of intracellular events during the early phase.

### PPE had little effect on C/EBPβ or C/EBPδ expressions and promoted IDM-induced mitotic clonal expansion in the early phase of adipogenesis

**Fig. 5:**
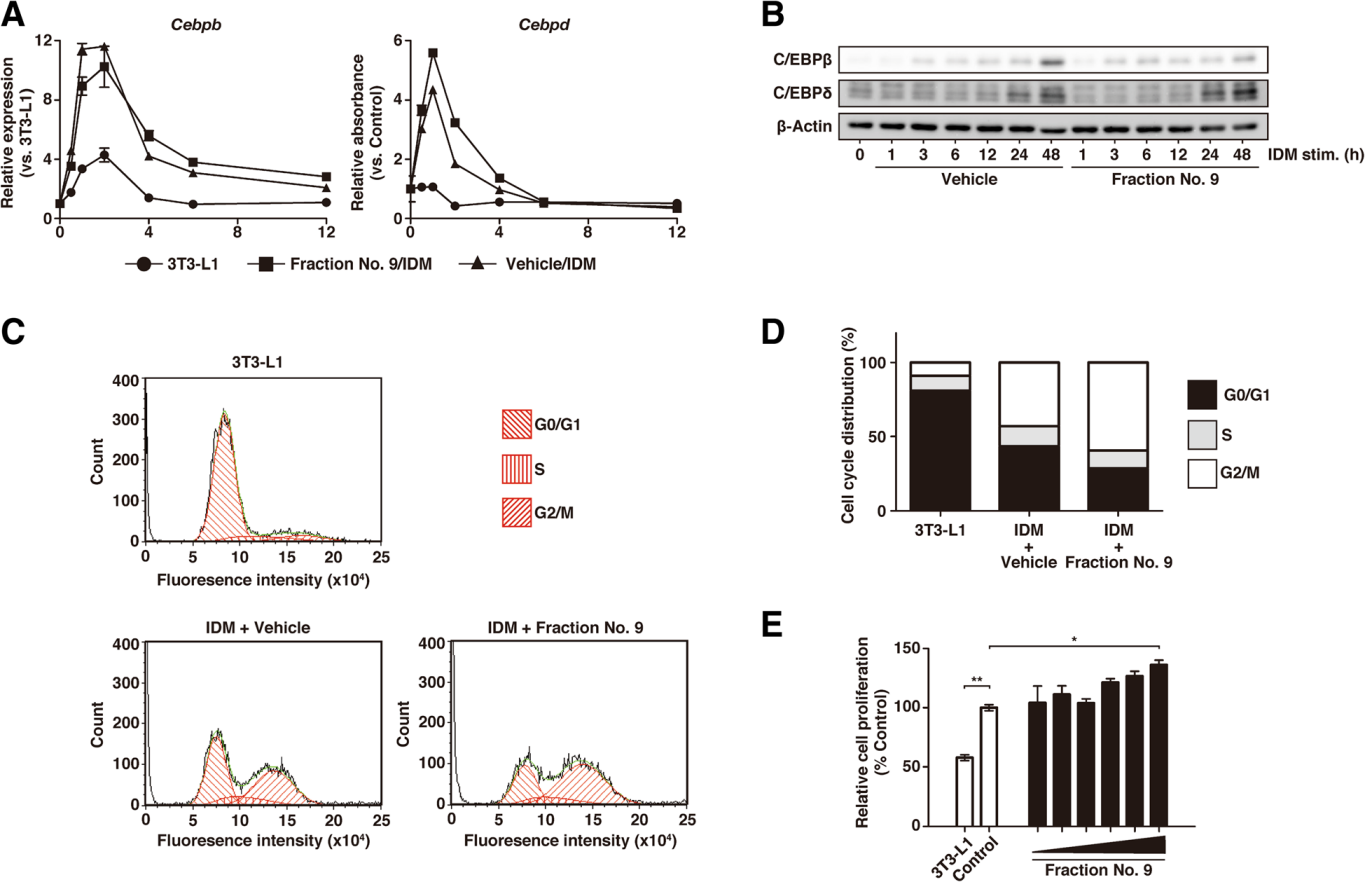
Effects of PPE on the expressions of C/EBPβ and C/EBPδ, and IDM-induced mitotic clonal expansion. **a** 3T3-L1 cells which reached confluence were cultured with fraction No. 9 and IDM for 12 h. The time-course (0, 0.5, 1, 2, 4, 6 and 12 h) of the gene expressions of C/EBPβ and C/EBPδ was analyzed by RT-qPCR. The gene expressions are presented relative to the value for *3T3-L1*. Experiments were performed in triplicate, and the data are presented as the mean ± SEM (n = 3). **b** 3T3-L1 cells which reached confluence were cultured with fraction No. 9 and IDM for 48 h. The time-course (0, 1, 3, 6, 12, 24 and 48 h) of the protein expressions of C/EBPβ and C/EBPδ was analyzed by western blotting. *Vehicle* represents cells cultured with PBS and IDM, and *3T3-L1* represents cells cultured without both fraction No. 9 and IDM. Experiments were repeated three times, and representative results are shown. **c** 3T3-L1 cells which reached confluence were cultured with IDM in the presence of fraction No. 9 (*IDM + Fraction No. 9*) or PBS (*IDM + Vehicle*) for 24 h. *3T3-L1* represents cells cultured without either fraction No. 9 or IDM for 24 h. The cells were stained with propidium iodide and then analyzed with flow cytometry. The experiments were repeated three times, and representative results are shown. **d** Bar diagram depicting the percentage of cells in different phases (G0/G1, S, G2/M) of the cell cycle. **e** 3T3-L1 cells which reached confluence were cultured for 48 h with IDM and a two-fold dilution series of fraction No. 9. *Control* represents cells cultured with only IDM, and *3T3-L1* represents cells cultured without either PPE or IDM. The cell proliferation data are presented as a percentage of the value in *Control* cells. Experiments were performed in triplicate, and the data are presented as the mean ± SEM (n = 3). *p < 0.05, **p < 0.01 vs. *Control*. Experiments were repeated at least three times, and representative results are shown

Because we observed that PPE suppressed PPARγ expression (Fig. 2 and 3f) and the suppression was due to the inhibition of differentiation during the early phase (Fig. 4b, c and d), we next examined the possibility that the expressions of C/EBPβ and C/EBPδ were altered by PPE. When we analyzed time-course changes in the gene expressions of C/EBPβ and C/EBPδ induced by IDM treatment with or without the addition of fraction No. 9 to 3T3-L1 cells, we found that fraction No. 9 had little effect on these gene expressions over the 12 h of culture (Fig. 5a). Similar results were also observed at the protein levels (Fig. 5b).

It is known that 3T3-L1 cells undergo approximately two rounds of mitotic clonal expansion (MCE) after IDM treatment, and recent studies have indicated that MCE at the early phase is a necessary step for terminal differentiation into mature adipocytes [38]. Since a previous report indicated that the first and second rounds of mitosis are completed at 24–36 h and 48–60 h, respectively, after IDM treatment [39], we conducted a flow cytometric analysis of the cell cycle in 3T3-L1 cells after treatment with PPE and IDM for 24 h. As shown in Fig. 5c and d, the G0/G1 population decreased from approximately 81% of 3T3-L1 cells cultured without IDM (*3T3-L1*) to approximately 44% of 3T3-L1 cells cultured with IDM and vehicle for 24 h (*IDM + Vehicle*), while the G2/M phase population increased from approximately 2% of the *3T3-L1* cells to approximately 43% of the *IDM + Vehicle* cells. On the other hand, as compared to the corresponding populations in *IDM + Vehicle* cells, the percentages of the G0/G1 and G2/M phase populations in 3T3-L1 cells that had been cultured with IDM and fraction No. 9 for 24 h (*IDM + fraction No. 9*) decreased and increased to approximately 29% and approximately 59%, respectively (Fig. 5c and d). We also examined the proliferation of differentiated 3T3-L1 cells using WST-8 and found that both PPE and fraction No. 9 promoted IDM-induced cell proliferation in a dose-dependent manner (Fig. 5e and Additional file 3: Figure S3).

### PPE suppressed 3T3-L1 adipogenesis via accelerated activation of p38 MAPK in the early phase

**Fig. 6:**
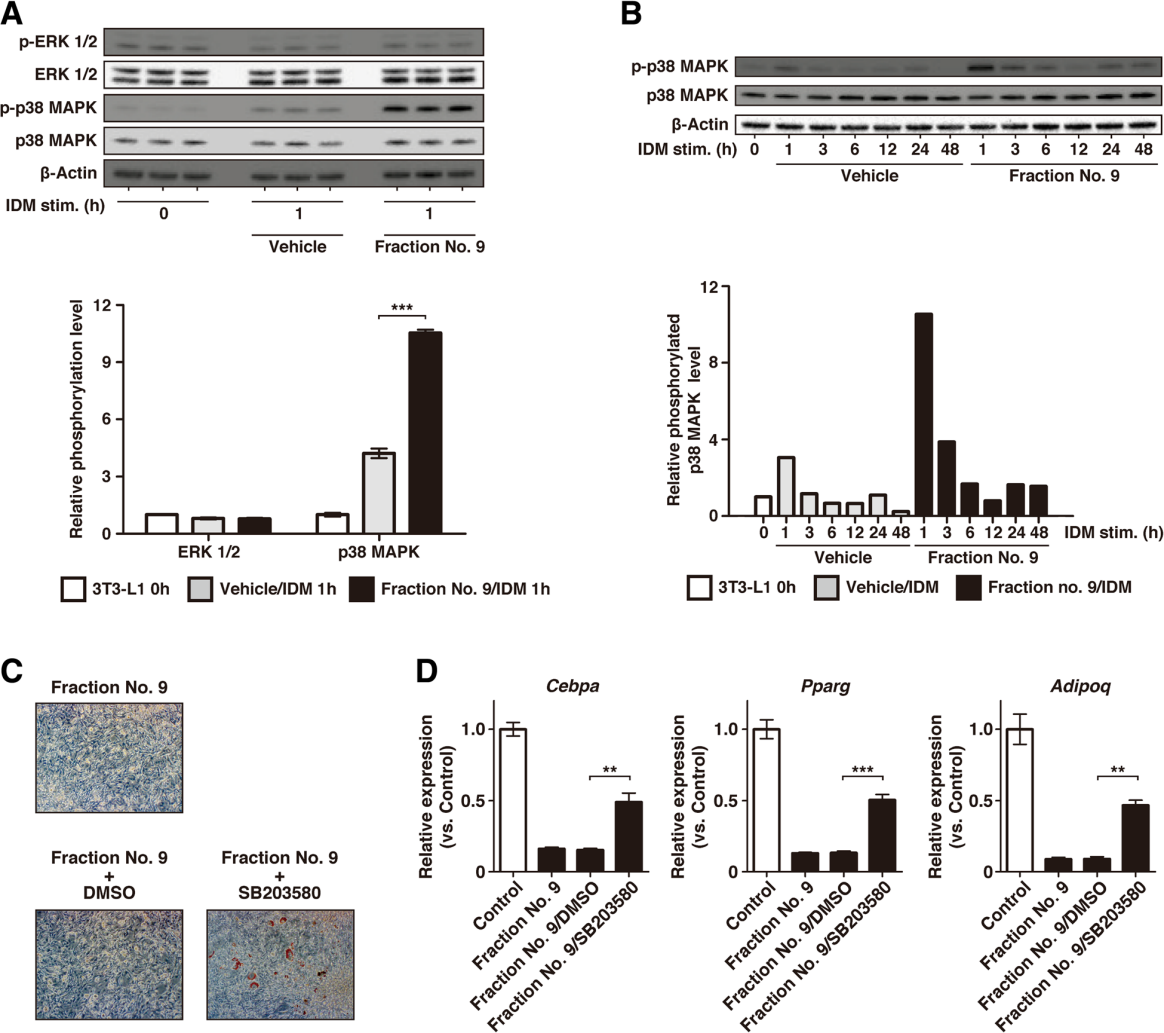
Effects of PPE on IDM-induced phosphorylation of MAPK. (A and B) 3T3-L1 cells which reached confluence were cultured with IDM in the presence of fraction No. 9 (*Fraction No. 9/IDM 1 h*) or PBS (*Vehicle/IDM 1 h*) for 24 h (**a**) or 0–48 h (**b**). The cell lysates were subjected to western blotting analysis with antibodies against phospho-p38 MAPK, total p38 MAPK, phospho-ERK1/2, total ERK1/2 or β-Actin. The relative intensity of each band of the phosphorylated forms after normalization for the levels in the total forms and β-Actin is shown as a bar graph. The experiment in (**a**) was performed in triplicate, and the data are presented as the mean ± SEM (n = 3). ***p < 0.005 vs. *Vehicle/IDM 1 h*. **c** 3T3-L1 cells which reached confluence were cultured with IDM for 2 days. The medium was changed every 48 h to fresh medium containing insulin until day 8. Fraction No. 9 was added during days 0–2, and 10 μM SB208035 or DMSO was also added during days 0–2 with 1 h of pre-treatment. After the cells were stained with Oil Red O, lipid droplets in the cells were imaged with a bright field microscope. **d** The gene expressions of adipocyte differentiation markers (*Cebpa*, *Pparg* and *Adipoq*) were analyzed on day 8 by RT-qPCR. *Control* represents cells cultured without fraction No. 9, and *Fraction No. 9* represents cells cultured with fraction No. 9. *Fraction No. 9/DMSO* and *Fraction No. 9/SB203580* represent cells cultured with fraction No. 9 in the presence of DMSO or 10 μM SB203580, respectively. The gene expression levels are presented as a ratio of the value in *Control* cells. Experiments were performed in triplicate, and the data are presented as the mean ± SEM (n = 3). ***p < 0.005 vs. *Control*

To elucidate the intracellular mechanisms underlying the suppression of adipogenesis by PPE, we focused on the effects of PPE on the IDM-induced proliferation of 3T3-L1 cells. Since it has been reported that ERK and p38 MAPK signaling control adipogenesis in both 3T3-L1 cells and mouse primary adipocytes [40–42], the IDM-induced phosphorylation states of ERK and p38 MAPK in 3T3-L1 cells were detected by western blotting. When we cultured 3T3-L1 cells with IDM for 1 h in the presence of fraction No. 9, the phosphorylated p38 MAPK was observed to be dramatically increased as compared with that in the presence of vehicle (Fig. 6a and b). Quite similar results were obtained by the treatment with PPE (Additional file 4: Figure S4). We also found that the suppression of the accumulation of lipid droplets (Fig. 6c) and gene expressions of adipocyte differentiation markers (*Cebpa*, *Pparg* and *Adipoq*) (Fig. 6d) by fraction No. 9 were partially inhibited by SB203580, a p38 MAPK inhibitor.

## Discussion

In this study, we found that porcine placental extract (PPE) suppressed 3T3-L1 adipogenesis by accelerating the activation of p38 MAPK, resulting in the suppression of lipid droplet accumulation. The differentiation of 3T3-L1 preadipocytes to mature adipocytes in the early phase (day 0 to day 2) is crucial for adipogenesis with PPARγ expression [39]. In the early phase, growth-arrested 3T3-L1 cells simultaneously reenter the cell cycle and undergo mitotic clonal expansion (MCE), and both these actions are dependent on CCAAT/enhancer-binding protein β (C/EBPβ) and C/EBPδ [43–45]. We showed that PPE scarcely attenuated MCE and the expressions of C/EBPβ and C/EBPδ during 3T3-L1 adipocyte differentiation in the early phase. These results suggest that PPE represses the PPARγ expression and adipogenesis without influencing the expression levels of C/EBPβ and C/EBPδ. In addition, treatment with PPE during the late phase (day 3 to day 8) had no effect on the accumulation of lipid droplets, suggesting that the effect of PPE was independent of glucose-uptake inhibition. In fact, PPE did not affect the phosphorylation levels of p70S6K, a kinase that acts downstream of insulin signaling, in 3T3-L1 cells when the cells were treated with insulin (data not shown).

The mitogen-activated protein kinases (MAPKs), which consist of extracellular signal-regulated kinase (ERK), c-jun N-terminal kinase (JNK) and p38 MAPK, are associated with proliferation, differentiation, mitosis, cell survival and death in various cells [46, 47]. Several studies have indicated that MAPK family members, including p38 MAPK, participate in adipogenesis [40, 48]. A recent study reported that p38 MAPK plays a negative role in adipogenesis via the inhibition of C/EBPβ and PPARγ transcriptional activities [42]. In support of this report, our results showed that the accelerated activation of p38 MAPK, but not ERK 1/2, in the early phase of 3T3-L1 differentiation suppressed the adipogenesis. On the other hand, some reports have shown that p38 MAPK plays a positive role in the 3T3-L1 adipogenesis [49–51]. Interestingly, the p38 MAPK-deficient and the inhibition of the p38 MAPK phosphorylation have been shown to increase the gene expressions of adipocyte differentiation markers [42, 52]. Moreover, inhibition of the p38 MAPK cascade over days 3 to 4 suppressed the expression of PPARγ, resulting in the repression of lipid-droplet accumulation [51, 53], whereas the accelerated activation of the p38 MAPK cascade during days 0 to 2 also suppressed the adipogenesis in this study. These observations suggest that p38 MAPK plays bidirectional roles in a differentiation phase-specific manner—i.e., promoting and inhibitory functions—in preadipocyte differentiation to mature adipocytes. Taken together, our results show that inhibition of p38 MAPK partially restored the attenuation of both the accumulation of lipid droplets and the gene expressions of adipocyte differentiation markers induced by PPE in our cellular model.

The placental extract is well known to enhance proliferation of skin fibroblasts [28]. However, it has not been determined which component(s) are responsible for the enhanced proliferation of fibroblasts. In this study, we showed that one of the substance in PPE that exhibits proliferative and suppressive activity against adipogenesis in 3T3-L1 preadipocytes was heat-stable. We also found that the substance could be separated by both ultrafiltration (< 3 kDa) and gel-filtration chromatography (fraction No. 9), indicating that it was a low-molecular-weight fraction. These results suggest that the active substance(s) derived from PPE were unlikely to be protein(s) or protein-containing macromolecule conjugate(s) which have a higher order structure, but were likely to be small molecules such as amino acids, nucleic acids, minerals, vitamins and hormones. We attempted further fractionation of fraction No. 9, which was separated by gel-filtration chromatography and exhibited a marked suppressive activity for adipogenesis, using an octadecylsilyl (ODS) column, and obtained a fraction that showed suppressive activity against adipogenesis (data not shown). However, the activity and yield loss were observed, suggesting that the deactivation by organic solvent occurred during the separation, and the separation methods need to be reconstructed. Limited information about the suppressive factors in PPE is available at present. These factors should be identified and characterized in future studies, which would likely provide mechanistic insights into adipogenesis and potential therapeutic strategies for obesity.

## Additional files

Additional file 1: **Figure S1.** The gene expression of brown/beige adipocyte differentiation markers, *Cidea*, *Pgc1a* and *Prdm16*, in 3T3-L1 cells cultured with PPE (1.0, 0.5 and 0.1 mg/mL) under differentiation conditions were analyzed on day 8 by RT-qPCR. *Control* represents cells cultured without PPE, and *3T3-L1* represents cells cultured without either PPE or differentiation-inducing agents. The gene expressions of *Pgc1a* and *Prdm16* in 3T3-L1 cells cultured with PPE are presented relative to the value in *3T3-L1* cells, and that of *Cidea* is presented relative to the value in *Control* cells. Experiments were performed in triplicate, and the data are presented as the mean ± SEM (*n* = 3). ND, not detected; *p < 0.05, ***p < 0.005 vs. *Control*. Experiments were repeated three times, and representative results are shown (TIF 1037 kb)

Additional file 2: **Figure S2.** (A) 3T3-L1 cells which reached confluence were cultured with or without each ultrafiltrated fraction, i.e., < 3 kDa or > 3 kDa separated from PPE using an Amicon, on a 12-well plate with IDM for 2 days. The medium was changed every 48 h to fresh medium containing insulin with or without each fraction until day 8. After the cells were stained with Oil Red O, lipid droplets in the cells were imaged with a bright field microscope. Each fraction was diluted with the culture medium at 1:100, 1/200 or 1/1000. (B) Stained lipid droplets were extracted with isopropanol and quantified by measuring the absorbance at 490 nm. Experiments were performed in triplicate, and data are presented as the mean ± SEM (*n* = 3). *p < 0.05, ***p < 0.005 vs. *Control*. Experiments were repeated at least three times, and representative results are shown (TIF 5665 kb)

Additional file 3: **Figure S3.** 3T3-L1 cells which reached confluence were cultured on a 96-well plate for 48 h with IDM and PPE (1.0, 0.5, 0.25, 0.125, 0.0625 and 0.03125 mg/mL). *Control* represents cells cultured with only IDM, and *3T3-L1* represents cells cultured without either PPE or IDM. The cell proliferation data are presented as a percentage of the value in *Control* cells. Experiments were performed in triplicate, and the data are presented as the mean ± SEM (n = 3). **p < 0.01, ***p < 0.005 vs. *Control*. Experiments were repeated at least three times, and representative results are shown (TIF 1124 kb)

Additional file 4: **Figure S4.** 3T3-L1 cells which reached confluence were cultured on a 12-well plate with IDM in the presence of PPE (*PPE/IDM 1 h*), fraction No. 9 (*Fraction No. 9/IDM 1 h*) or PBS (*Vehicle/IDM 1 h*) for 24 h. Cells were then lysed in 1 × SDS sample buffer, and the cell lysates were subjected to western blotting analysis with antibodies against phospho-p38 MAPK, total p38 MAPK or β-Actin. The relative intensity of each band of the phosphorylated forms after normalization for the levels in the total forms and β-Actin is shown as a bar graph. The experiments were performed in triplicate, and the data are presented as the mean ± SEM (n = 3). **p < 0.01 vs. *Vehicle/IDM 1 h*. Experiments were repeated at least three times, and representative results are shown. (TIF 1742 kb)

## Abbreviations

ANOVA: Analysis of variance
C/EBP: CCAAT/enhancer-binding protein
DEX: Dexamethasone
EGCG: Epigallocatechin gallate
ERK: Extracellular signal-regulated kinase
FBS: Fetal bovine serum
GTC: Green tea catechins
IBMX: 3-isobutyl-1-methylxanthine
JNK: c-jun N-terminal kinase
MAPK: Mitogen-activated protein kinase
MCE: Mitotic clonal expansion
NASH: Non-alcoholic steatohepatitis
NBCS: Newborn calf serum
ODS: Octadecylsilyl
PBS: Phosphate-buffered saline
PPAR: Peroxisome proliferator-activated receptor
PPE: Porcine placental extract
RT-qPCR: Reverse transcription-quantitative polymerase chain reaction
SDS-PAGE: Sodium dodecyl sulfate-polyacrylamide gel electrophoresis
WST: Water-soluble tetrazolium salts
