# Supplementary figures and images for: Placental extract suppresses differentiation of 3T3-L1 preadipocytes to mature adipocytes via accelerated activation of p38 MAPK during the early phase of adipogenesis
Source: Nutr Metab (Lond). 2019 May 20;16:32. doi: 10.1186/s12986-019-0361-8 (PMC6528359; doi:10.1186/s12986-019-0361-8)

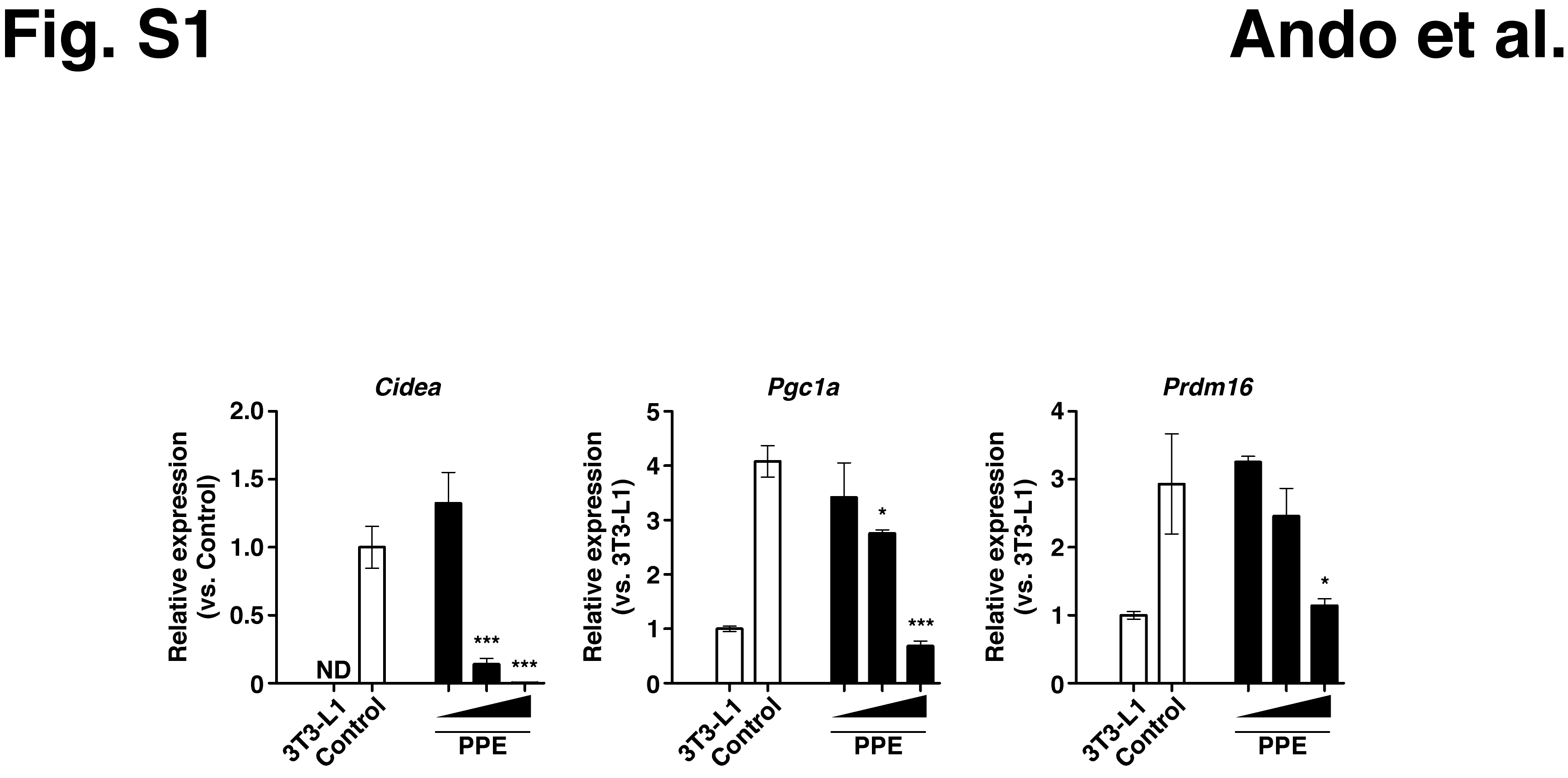

Supplement: Supplementary file 1 — Figure S1. The gene expression of brown/beige adipocyte differentiation markers, Cidea, Pgc1a and Prdm16, in 3T3-L1 cells cultured with PPE (1.0, 0.5 and 0.1 mg/mL) under differentiation conditions were analyzed on day 8 by RT-qPCR. Control represents cells cultured without PPE, and 3T3-L1 represents cells cultured without either PPE or differentiation-inducing agents. The gene expressions of Pgc1a and Prdm16 in 3T3-L1 cells cultured with PPE are presented relative to the value in 3T3-L1 cells, and that of Cidea is presented relative to the value in Control cells. Experiments were performed in triplicate, and the data are presented as the mean ± SEM (n = 3). ND, not detected; *p < 0.05, ***p < 0.005 vs. Control. Experiments were repeated three times, and representative results are shown (TIF 1037 kb) [file 12986_2019_361_MOESM1_ESM.tif]

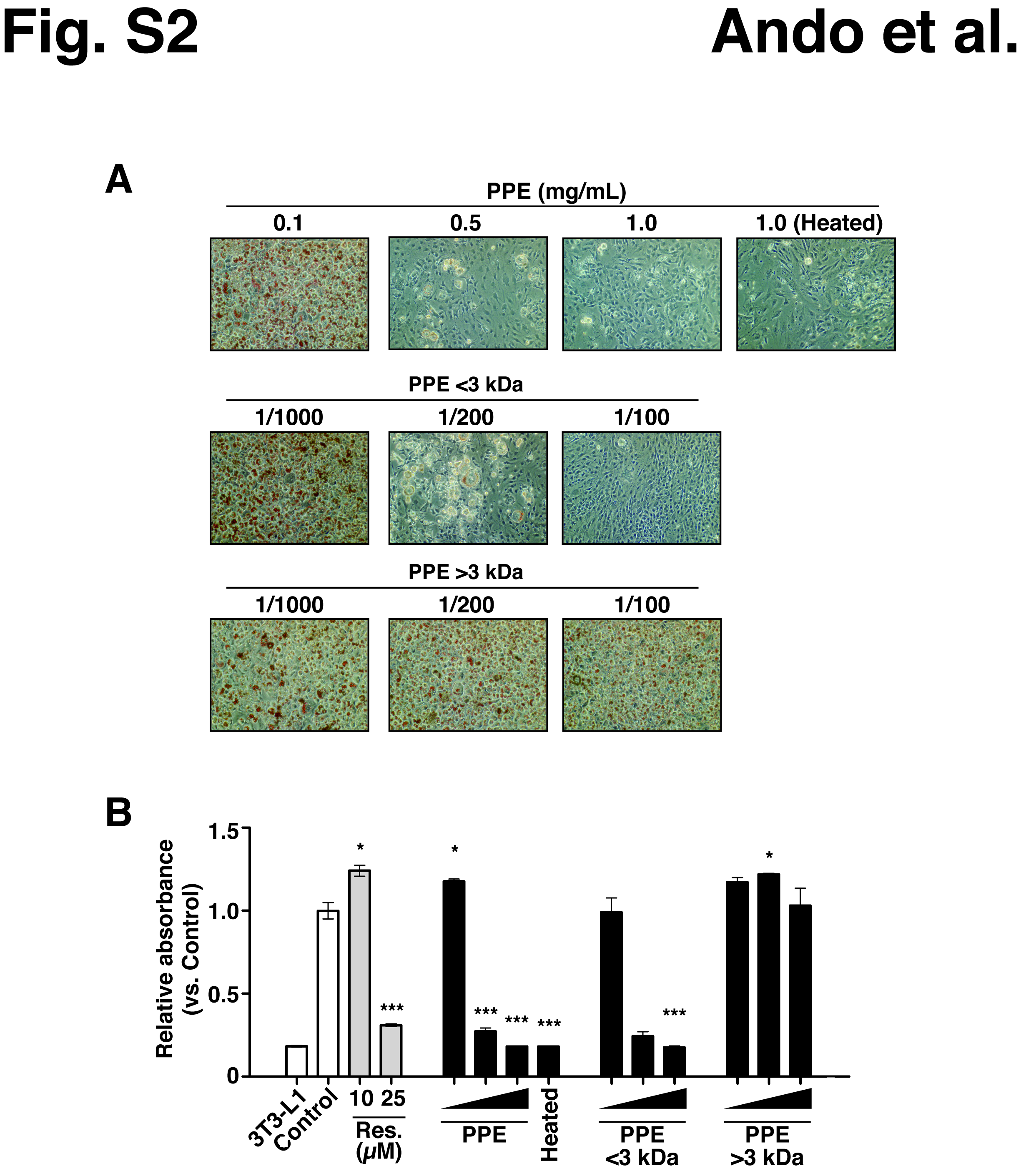

Supplement: Supplementary file 2 — Figure S2. (A) 3T3-L1 cells which reached confluence were cultured with or without each ultrafiltrated fraction, i.e., < 3 kDa or > 3 kDa separated from PPE using an Amicon, on a 12-well plate with IDM for 2 days. The medium was changed every 48 h to fresh medium containing insulin with or without each fraction until day 8. After the cells were stained with Oil Red O, lipid droplets in the cells were imaged with a bright field microscope. Each fraction was diluted with the culture medium at 1:100, 1/200 or 1/1000. (B) Stained lipid droplets were extracted with isopropanol and quantified by measuring the absorbance at 490 nm. Experiments were performed in triplicate, and data are presented as the mean ± SEM (n = 3). *p < 0.05, ***p < 0.005 vs. Control. Experiments were repeated at least three times, and representative results are shown (TIF 5665 kb) [file 12986_2019_361_MOESM2_ESM.tif]

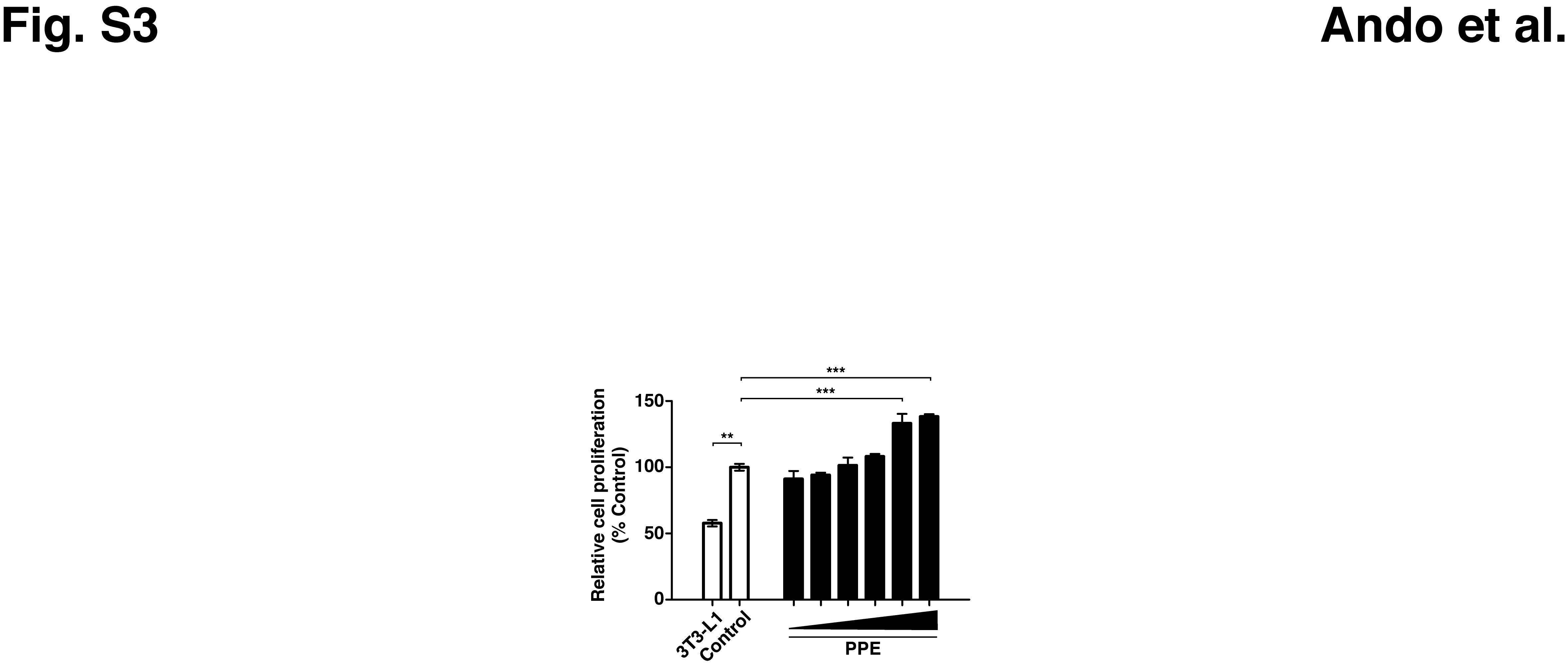

Supplement: Supplementary file 3 — Figure S3. 3T3-L1 cells which reached confluence were cultured on a 96-well plate for 48 h with IDM and PPE (1.0, 0.5, 0.25, 0.125, 0.0625 and 0.03125 mg/mL). Control represents cells cultured with only IDM, and 3T3-L1 represents cells cultured without either PPE or IDM. The cell proliferation data are presented as a percentage of the value in Control cells. Experiments were performed in triplicate, and the data are presented as the mean ± SEM (n = 3). **p < 0.01, ***p < 0.005 vs. Control. Experiments were repeated at least three times, and representative results are shown (TIF 1124 kb) [file 12986_2019_361_MOESM3_ESM.tif]

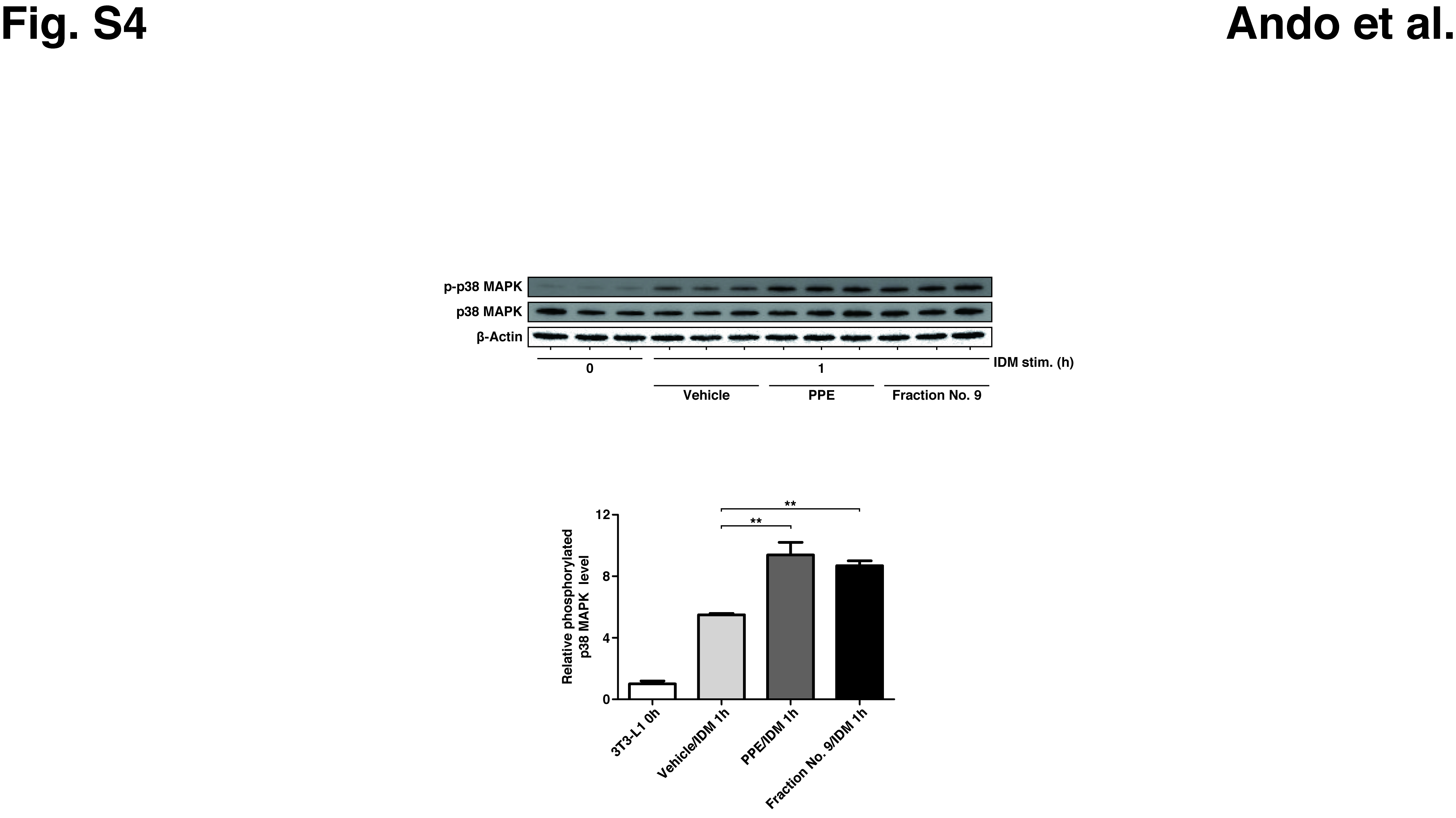

Supplement: Supplementary file 4 — Figure S4. 3T3-L1 cells which reached confluence were cultured on a 12-well plate with IDM in the presence of PPE (PPE/IDM 1 h), fraction No. 9 (Fraction No. 9/IDM 1 h) or PBS (Vehicle/IDM 1 h) for 24 h. Cells were then lysed in 1 × SDS sample buffer, and the cell lysates were subjected to western blotting analysis with antibodies against phospho-p38 MAPK, total p38 MAPK or β-Actin. The relative intensity of each band of the phosphorylated forms after normalization for the levels in the total forms and β-Actin is shown as a bar graph. The experiments were performed in triplicate, and the data are presented as the mean ± SEM (n = 3). **p < 0.01 vs. Vehicle/IDM 1 h. Experiments were repeated at least three times, and representative results are shown. (TIF 1742 kb) [file 12986_2019_361_MOESM4_ESM.tif]
